# Diversity and structural characteristics of soil microbial communities in different habitats of wild *Lilium regale* Wilson in Wenchuan area

**DOI:** 10.1080/21655979.2021.1997366

**Published:** 2021-12-09

**Authors:** Jie Xie, Ze Wu, Xiaoyu Zhang, Tong Peng, Chunmei Yang, Jianjun Zhang, Jian Liang

**Affiliations:** a College of Life Sciences, Sichuan Normal University, Chengdu, P.R. China; b National alcohol and processed food quality supervision and Inspection Center, Chengdu Institute of Product Quality Inspection Co.,Ltd, Chengdu, 610041, P.R. China; c Institute of Horticulture, Chengdu Academy of Agriculture and Forestry Sciences, Chengdu, 611130, P.R. China; d College of Resources and Environment, Aba Teachers University, Wenchuan, China

**Keywords:** High-throughput sequencing, microbial community structure, diversity, *L.regale*, rhizosphere microorganisms, soil factors

## Abstract

*Lilium regale* Wilson (*L.regale*), originated in the Minjiang River basin in Sichuan, China, has different phenotypic characteristics in different environments. To analyze the correlation between the phenotypes of *L.regale* and its soil micro-ecological environment, wild habitat soil of *L.regale* at the two altitudes were selected to analyze the diversity and community structure of microorganisms in soil, and measure the soil physicochemical factors and enzyme activities. The structural composition and diversity of fungal and bacterial communities in hillside and valley soils were significantly different (*p*< 0.01). Soil available potassium (AK) and soil enzyme activities such as urease (S_UE), sucrase (S_SC), and catalase (S_CAT) differed significantly different between hillsides and valleys (*p* < 0.01), while organic matter (OM), total phosphorus (TP), and polyphenol oxidase (S_PPO) had no great variances. Correlation analysis was conducted between the common and differential microorganisms and the morphological characteristics, soil physicochemical factors and soil enzyme activities of *L.regale* in both hillside and valley. The results showed that both of the fungal and bacterial could be clustered into two distinct groups by positive and negative correlations, suggesting that the representative microorganism may have structural characteristics that are directly related to soil physicochemical properties and enzyme activities, which conversely affect the phenotype of Lily. Therefore, the study on the native species of horticultural plants and the local soil microhabitat environment will benefit the conservation of wild Lily and provide theoretical guidance for the domestication and breeding of horticultural plants.

## 1 Introduction

‘China, Mother of Gardens’, Ernest H. Wilson referred to Sichuan, China, as the mother of gardens. The tall, gorgeous and fragrant *Lilium regale* Wilson (*L.regale)* was introduced to England by Wilson in 1908, saving most European native Lily species from extinction caused by the Lily virus. According to cross robust *L.regale* with European Lily by European horticulturists, many new varieties of Lily that were adaptable and highly resistant to viruses appeared [1]. *L.regale* is the female parent of most fresh-cut Lily varieties in the world, and most Lily contain its shadow. Because of the large and fragrant flowers, *L.regale* plays a keystone status in Lily breeding. In addition, multiple ecological functions of *L.regale* in special habitats are prominent, such as protecting the mountain, slowing down scouring, and improving landscape quality.

Plants are colonized by a multitude of micro-organisms, collectively called ‘microbiome,’ playing essential roles in the niches they inhabit. Soil microbiome are the most active part of the soil ecosystem closely related to the development of plants. They participate in a variety of biochemical reactions and they are crucial to the nutrient cycling in the soil. They also influence above-ground ecosystems by contributing to plant nutrition, health, soil structure and fertility [2,3]. Therefore, the soil microbial community is an important biological indicator of soil functions [4,5]. High-throughput sequencing is used extensively to study the correlation between soil microbial communities and plant traits and biomass [6,7]. Shang showed that the main difference between healthy and wilted Lily samples in Lanzhou was microbial composition and functional diversity, which was closely related to the plant health status [8]. When Lily and maize are intercropped, the soil micro-environment is altered, having an effect on the diversity and structure of the Lily rhizosphere microbial community and Lily yield [9]. Yan Zhang et al. explored the characteristics of the rhizosphere microbial community of blueberry and analyzed the beneficial and core microorganisms of blueberry, and found that it is beneficial to the health and production of blueberry based on the microbial community [10].

Wenchuan area, a typical arid river valley in the upper Minjiang River, is dominated by high mountain and canyon landscapes with abundant environmental variation, which makes *L.regale* show differences in phenotype [11] and biomass distribution [12]. Cheng et al. found that *L.regale* in different habitats differed significantly in morphological and physiological characteristics, especially in basal diameter, plant height, leaf length, and leaf width [13]. Therefore, systematic research on soil microbial ecology is important for the domestication and breeding of *L.regale*. Despite its crucial role, relevant studies on the soil microbiological environment of *L.regale* habitats are currently insufficient. In this study, we respectively investigated the community diversity and structure of soil microorganisms from the representative wild *L.regale* in Wenchuan area, hillsides and valleys, and elucidated their interactions with the spatial distribution patterns of soil physicochemical factors and soil key metabolic enzymes. Combining the morphological and physiological characteristics of *L.regale* in different habitats, we aimed at exploring the close relationship between the soil micro-ecological environment and phenotypes of *L.regale*. These findings may provide a scientific and theoretical guidance to the conservation, domestication and breeding of wild *L.regale* resources.

## 2 Materials and methods

### 2.1 Study area and soil sampling

Wenchuan area is located in the semi-arid valley of the upper reaches of the Minjiang River at the northwestern edge of the Sichuan basin (*E* 102°51ʹ~103°44ʹ, *N* 30°45ʹ~31°43ʹ), which belongs to the warm temperate continental semi-arid monsoon climate, with low and stable precipitation, uneven daily maximum precipitation due to seasonal distribution. The dry and rainy seasons are different, with obvious winter dryness, and frequent spring droughts and summer droughts [14]. The average annual temperature is 13.5°C ~ 14.1°C, rainfall is 528.7 ~ 1332.2 mm, annual sunshine is 1693.9 ~ 1042.2 hours, and the frost-free period is 247 ~ 269 days.

**Figure 1.**
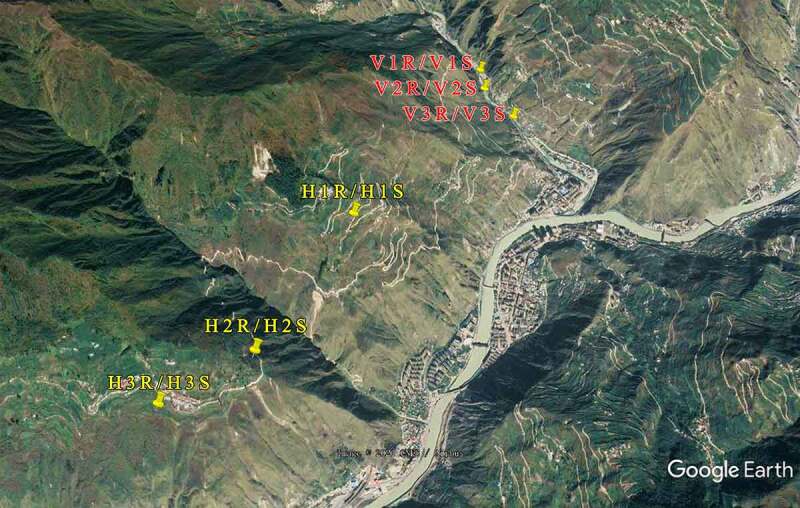
Schematic diagram of sampling point location

Two natural growing areas of wild *L.regale* were selected in the Wenchuan area: one is in the hillside and the other is in the river valley. The altitudes are about 1900–2000 m and 1200–1400 m separately (Figure 1). Three concentrated populations of *L.regale* were randomly selected at the sampling sites, and five *L.regale* plants in suitable growth condition were selected for each population by the five-point sampling method. The rhizosphere soil was collected through the root shaking method, while non- rhizosphere soil was collected around the root system with a clean auger about 30 cm away from the sampled plant and at a depth of 20 cm. All samples were stored in sterile bags and marked, then brought back to the laboratory with a low-temperature sampling box as soon as possible. Each soil sample from the same population was mixed in equal amounts as one test sample, for a total of 12 samples (Table 1). The soil samples were divided into 2 parts, one was stored in an ultra-low temperature refrigerator at −80°C for microbial community characterization, and the other was air-dried, de-hybridized, ground and sieved for analysis of soil physicochemical factors and enzyme activities.

**Table 1.** Sampling information of soil samples from wild *Lilium regale* Wilson habitat

Smples	Habitat type	Longitude	Latitude	Altitude (m)
H1R	Hillside	103°33ʹ47.59” *E*	31°28ʹ38.26” *N*	2047
H2R	Hillside	103°33ʹ21.65” *E*	31°28ʹ0.34” *N*	1992
H3R	Hillside	103°32ʹ56.26” *E*	31°27ʹ47.37” *N*	1997
H1S	Hillside	103°33ʹ47.59” *E*	31°28ʹ38.26” *N*	2047
H2S	Hillside	103°33ʹ21.65” *E*	31°28ʹ0.34” *N*	1992
H3S	Hillside	103°32ʹ56.26” *E*	31°27ʹ47.37” *N*	1997
V1R	Valley	103°34ʹ32.20” *E*	31°29ʹ45.75” *N*	1385
V2R	Valley	103°34ʹ33.50” *E*	31°29ʹ38.08” *N*	1357
V3R	Valley	103°34ʹ43.59” *E*	31°29ʹ26.89” *N*	1350
V1S	Valley	103°34ʹ32.20” *E*	31°29ʹ45.75” *N*	1385
V2S	Valley	103°34ʹ33.50” *E*	31°29ʹ38.08” *N*	1357
V3S	Valley	103°34ʹ43.59” *E*	31°29ʹ26.89” *N*	1350

HR:Hillside rhizosphere soils; HS:Hillside non- rhizosphere soils; VR:Valley rhizosphere soils; VS:Valley non- rhizosphere soils.

### 2.2 DNA extraction, amplification and Mi-Seq sequencing

Genomic DNA was extracted directly by a soil DNA extraction kit (Omega Bio-Tek, GA, USA) according to the manufacturer’s instruction. The concentration and purity of DNA extracts were determined by a NanoDrop ND2000C spectrophotometer (Thermo Scientific, Wilmington, USA). Qualified total genomic DNA was amplified using an ITS1F/ITS2R primer set, which amplifies the ITS1 region of the internal transcribed spacer, and a 338 F/806 R primer set, which amplifies the V3-V4 region of the 16S rDNA gene, to determine the diversity and compositions of the bacterial and fungal communities in each sample [15,16]. The samples were sequenced on an Illumina MiSeq platform at Majorbio Bio-pharm Technology Co. Ltd (Shanghai, China). Complete data sets in this study have been deposited in the National center of Biotechnology Information (NCBI) Sequence Read Archive database with the project number PRJNA707142.

### 2.3 Soil physiochemical analysis

Soil organic matter (OM) was obtained by a mixture of potassium dichromate under heating as described by Yeomans [17]. Total nitrogen (TN) was determined based on Kjeldahl method [18]. Total potassium (TK) and available potassium (AK) were measured by flame photometer [19]. Total phosphorus (TP) and available phosphorus (AP) were respectively determined using molybdenum antimony anti colorimetry and spectrophotometry [20].

### 2.4 Soil enzyme activity analysis

Soil urease (S_UE) activity was determined by sodium phenol sodium hypochlorite colorimetry [21]. The activity of soil sucrase (S_SC) and soil cellulase (S_CL) was measured by 3,5-Dinitrosalicylic acid colorimetry [22]. Soil polyphenol oxidase (S_PPO) activity and soil catalase (S_CAT) activity was determined by pyrogallol colorimetry and ultraviolet spectrophotometry respectively [23].

### 2.5 Data processing and analysis

Bioinformation analysis of sequencing data was performed using Meijicloud online analysis system (https://cloud.majorbio.com). The PE reads obtained from Miseq sequencing were first spliced with Flash software, and then the sequences were quality-controlled and filtered with Fastp software to obtain valid sequences. The sequences were clustered by OTUs (Operational Taxonomic Units) according to 97% similarity level using Uparse software. Species taxonomic annotation of OTUs at 97% similarity level was performed using RDP Classifier software. Biodiversity indices such as Chao index, Ace index, Shanon index, Simpson index and Average index were calculated using Mothur software, and species abundance tables at each taxonomic level were generated utilizing Qiime software.

The statistical analyses were conducted with SPSS 19.0, and then plotted with Origin Pro 2021. The Spearman’s rank correlation coefficient using R software was applied to investigate the correlation between soil microbiome and environmental properties.

## 3 Results

*L.regale* has different phenotypic characteristics in different habitats in Wenchuan area. To identify the influence of soil micro-ecological environments on the phenotypes of *L.regale*, the inter-rhizosphere and non-rhizosphere soil were respectively selected from the representative wild *L.regale* in Wenchuan area, hillsides and valleys. Combined with the spatial distribution patterns of soil physicochemical factors and soil key metabolic enzymes, the community structure and diversity characteristics of soil microorganisms in *L.regale* habitats in Wenchuan area were analyzed, to elucidate the interactions with phenotypes of *L.regale*. The detailed results of the study show as following:

### 3.1 Comparison of phenotypic characteristics of L.regale

**Figure 2.**
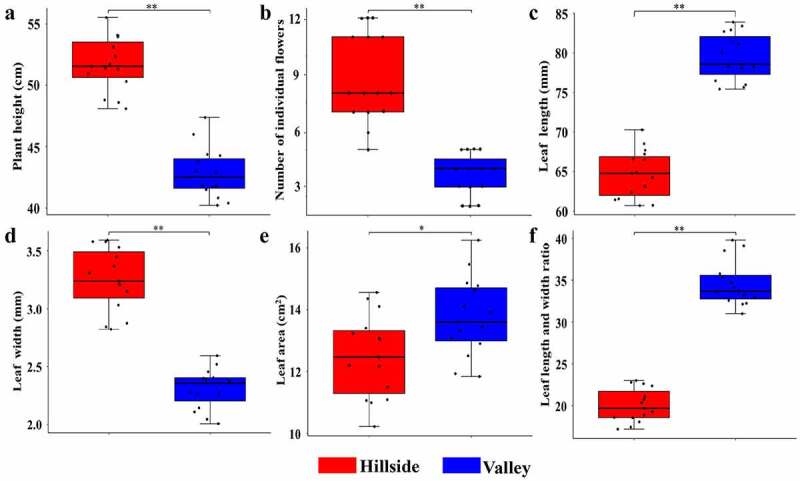
Comparison of phenotypic characteristics of *L.regale.*

Growth morphological indexes of Lily plants on hillside and river valleys such as plant height, leaf length, leaf width, aspect ratio, and flower number were measured respectively, the results are shown in Figure 2. There were great differences in the appearance status of *L.regale* on the hillsides and river valleys. Almost all Lily plants on hillsides were above 50 cm in height, which were significantly higher than that on river valleys (p < 0.01) ranged 40 cm from 44 cm. The number of flowers of Lily on hillsides (some up to 12 flowers) was much more than that in river valleys as few as 2 flowers (*p* < 0.01). Compared to the Lily on hillside, the leaf length and leaf aspect ratio of Lily in the river valley were significantly larger (*p* < 0.01), but the leaf width was much smaller (*p* < 0.01). In addition, the great majority of leaf surface of Lily in the valley was mostly lance-shaped, while that of Lily on hillside mostly presented oval or strip-shaped. Therefore, phenotypic characteristics of wild *L.regale* in different habitats differed significantly.

### 3.2 Soil microbial community diversity

**Figure 3.**
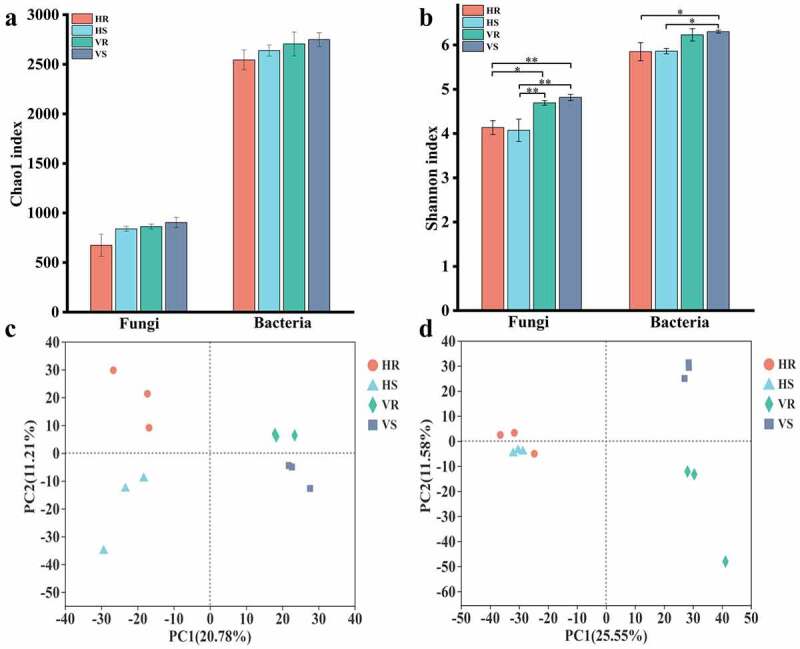
Comparison of soil microbial community richness and diversity in *L.regale* habitat and principal component analysis. (a) chao1 index; (b) shannon index; (c) Fingi; (d) bacterial

A total of 750,211 effective sequences of fungi were obtained in 12 soil samples, and the average number of sequences in HR, HS, VR and VS were 56,650, 61,684, 69,624 and 61,931, respectively, with an average length of 239 bp. 753,679 effective sequences of bacteria were obtained, and the average number of sequences in HR, HS, VR and VS were 50,219, 55,085, 87,870 and 58,050, and the average length of the sequences was 414 bp. The sum of 2622 fungal OTUs and 4262 bacterial OTUs were obtained by QC and filtering at 97% sequence similarity level. Alpha diversity analysis showed that the Chao1 index of fungi or bacteria did not differ significantly among groups, indicating that there was no considerable difference in the abundance of fungi or bacteria in soils of different habitats. However, the Chao1 index of bacteria was greater than that of fungi (*p* < 0.01), indicating that the abundance of bacteria in the soil was much higher than that of fungi (Figure 3(a)). The Shannon index varied significantly among groups, with the Shannon index of fungi on hillside which was much lower than that of river valleys (*p* < 0.05), and the Shannon index of bacteria on hillside also differed from river valley samples, indicating that the diversity of fungi or bacteria in soils of different habitats differed. However, there was no significant difference in the diversity of fungi or bacteria between inter- and non- rhizosphere soils in the same area (Figure 3(b)).

Beta diversity analysis by using principal component analysis for samples from hillsides and river valleys showed that both of hillside soil samples (HR and HS) and river valley soil samples (VR and VS) for fungi or bacteria were on the positive and negative half axes of the PCA1 axis and far apart, respectively, indicating that the community composition structure of fungi or bacteria differed greatly in soils at different elevations (Figure 3(c,d)). Analysis of inter- and non- rhizosphere samples from the same elevation showed that samples from HR and HS were farther apart, while samples from VR and VS were closer together, suggesting that the structural differences in fungal communities were large in hillside inter- and non- rhizosphere soils, while they were smaller in river valley inter- and non- rhizosphere soils (Figure 3(c)). In contrast to the composition of fungi, that of bacteria in inter- and non- rhizosphere soils differed less on hillsides and more in river valleys (Figure 3(d)). Thus, the structural composition of soil microorganisms differed considerably in different habitats, which may be related to the difference in altitude.

### 3.3 Soil microbial community composition

#### 3.3.1 Analysis of community composition at the phylum level

**Figure 4.**
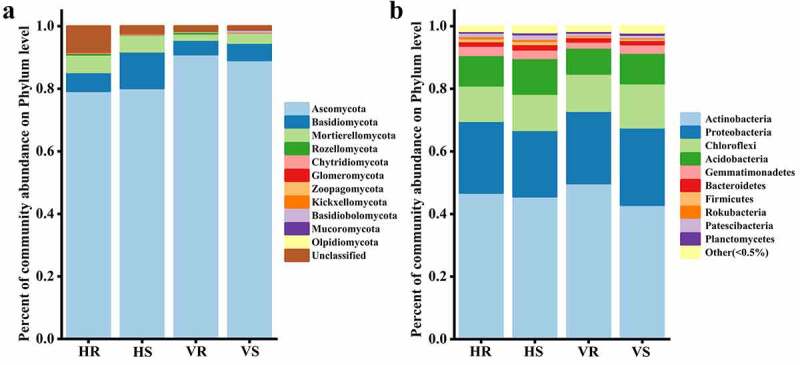
The composition of soil microbial community at phylum level. (a) fungi; (b) bacteria

The composition and relative abundance of microbial communities at the phylum level for the 12 soil samples are shown in Figure 4. The fungi co-occurring in HR, HS, VR and VS were: *Ascomycota* (79.28%-91.00%), *Basidiomycota* (4.67%-11.80%), *Mortierellomycota* (1.98%-5.61%), *Rozellomycota* (0.08%-0.55%) and *Chytridiomycota* (0.11%-0.33%). *Ascomycota* was the dominant group in the soil of *L.regale* habitat, and its relative abundance was less on the hillside (79.28%-80.14%) than in the valleys (89.16%-91.01%). In addition, *Zoopagomycota* and *Basidiobolomycota* were the different fungi between the two habitat soils, *Zoopagomycota* (0.038%-0.056%) only present in hillside soil and *Basidiobolomycota* (0.063%-0.54%) only present in river valley soil (Figure 4(a)). It can be seen that the relative abundance of the fungal community groups differed somewhat at the phylum level, but the differences were not significant.

Among the bacteria, *Actinobacteria* (42.88%-49.77%), *Proteobacteria* (21.23%-24.73%), *Chloroflexi* (11.27%-14.10%), *Acidobacteria* (8.37%-11.39%), *Gemmatimonadetes* (1.90%-2.97%), *Bacteroidetes* (1.38%-1.75%), *Patescibacteri* (0.82%-1.48%), *Firmicutes* (0.43–0.98%), *Rokubacteria* (0.16%-0.73%), *Planctomycetes* (0.48%-0.68%) and *Verrucomicrobia* (0.40%-0.68%) had high relative abundance (relative abundance >0.5%) (those with relative abundance less than 0.5% in all groups were classified as Other) (Figure 4(b)). Similar to the fungal community, the relative abundance of the bacterial community groups did not differ much at the phylum level.

#### 3.3.2 Analysis of community composition analysis at the genus level

**Figure 5.**
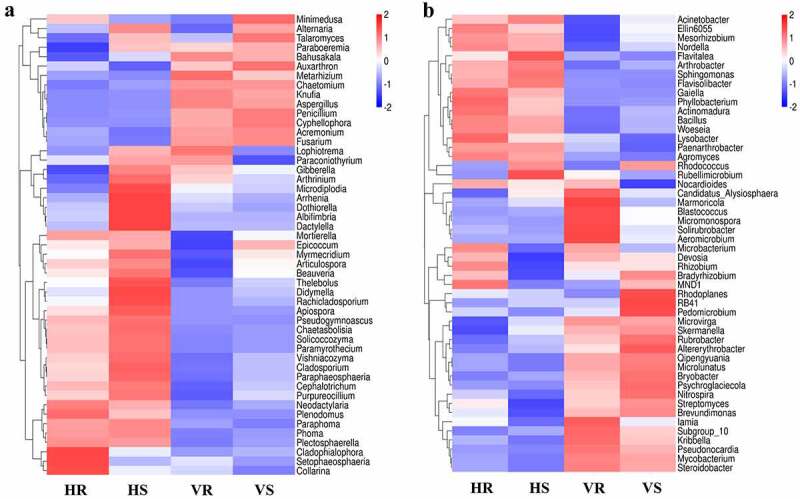
The composition of soil microbial community at genus level. (a) fungi; (b) bacteria

The community composition and abundance of high-abundance species in HR, HS, VR and VS were analyzed at the genus level, as shown in Figure 5. For each group of microorganisms by the relative abundance of 1% and above, there were 27 dominant fungal genera and 14 dominant bacterial genera. Among them, the genera *Mortierella* (1.15%-5.61%), *Acremonium* (2.24%-3.22%), *Gibberella* (1.61%-7.04%), *Penicillium* (1.39%-9.17%), *Arthrobacter* (4.98%-15.1%), *Blastococcus* (1.46%-4.61%), *Solirubrobacter* (1.92%-3.74%), Ellin6055 spp. (1.86%-2.67%) and RB41 spp. were the common genera in hillside and valley soils, with relative abundance greater than 1% in each group. There were still different fugal genera present in the soils of the two sites, and they were *Solicoccozyma* (H:4.35%-9.8%, V:0.66%-0.81%), *Cladosporium* (H:4.00%-13.99%,V:0.59%-1.29%), *Paraphoma* (H:2.00%-2.47%, V:0.17%-0.24%), *Penicillium* (H:1.39%-1.51%,V:6.34%-9.17%), *Metarhizium* (H:0.55%-0.71%,V:3.54%-9.35%), *Fusarium* (H:0.33%-0.5%,V:3.29%-3.77%), *Auxarthron* (H:0.12%-0.45%,V:1.51%-4.00%), *Knufia* (H:0.35%,V:2.14%-2.75%), *Chaetomium* (H:0.21%-0.31%,V:2.14%-2.18%), *Aspergillus* (H:0.051%,V:3.39%-5.73%), *Bahusakala* (H:0.047%-0.26%,V:1.11%-1.74%), and *Coniosporium* (H:0.004%-0.011%,V:1.09%-1.14%). The differential bacterial genera were *Arthrobacter* (H:12.45-12.1%, V:4.98%-6.08%), *Sphingomonas* (H:1.11%-1.25%, V:0.65%-0.66%), *Gaiella* (H:1.92%-2.90%, V: 0.56%-0.58%), *Crossiella* (H:0.10%, V:2.09%-2.12%), *Mycobacterium* (H:0.78%-0.89%, V:1.59%-1.84%), *Microvirga* (H:0.74%-0.99%, V:1.34%-1.38%), and *Rubrobacter* (H:0.54%-0.79%, V:1.23%-1.85%). In conclusion, the fungal and bacterial community composition of *L.regale* in hillside and valley soils are significantly different at the genus level compared to the phylum level.

### 3.4 Soil physicochemical factors and enzyme activities

**Figure 6.**
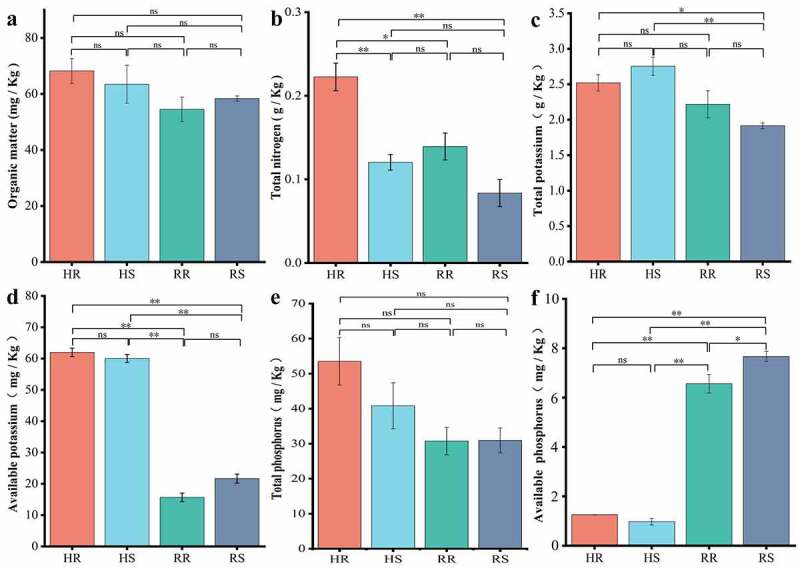
Physical and chemical properties of soil in different habitats of *L.regale.*

The results of soil physicochemical factor analysis are presented in Figure 6. The nutrients in each group of soils were at very low levels except for available potassium (AK), which was at a medium level [24]. A comparison of physicochemical factors between hillside and river valley soils revealed that all factors differed from the two sites, except for organic matter (OM) and total phosphorus (TP), which were not different significantly. Among these factors, the content of available potassium (AK) was extremely significant higher in hillsides than in river valleys (*p*< 0.01), while the content of available phosphorus (AP) was extremely significant higher in river valleys than in hillsides (*p* < 0.01). The differences in physicochemical factors between inter- and non- rhizosphere soils in the same area were small. Therefore, there were differences in physicochemical properties between hillside and river valley soils.

**Figure 7.**
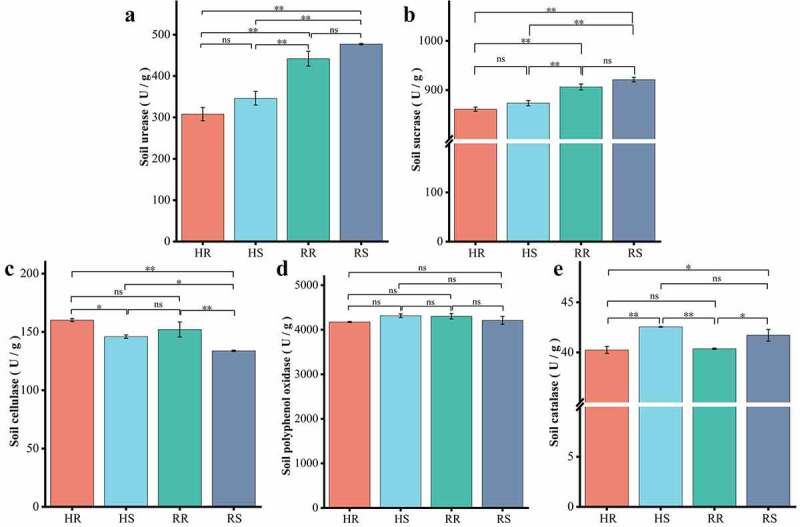
Soil enzyme activities in different habitats of *L.regale* (U/g)

The results of soil enzyme activities are shown in Figure 7. The activity of polyphenol oxidase (S_PPO) in soil was the highest, and there was no significant difference between the hillside and the valley. Activities of urease (S_UE) and sucrase (S_SC) in the soil differed extremely significantly (*p* < 0.01) between hillsides and valleys. Cellulase (S_CL) and catalase (S_CAT) activities were significantly different between inter- and non- rhizosphere, and the activity of catalase (S_CAT) in non- rhizosphere was greater than that in inter-rhizosphere (*p* < 0.05), while soil cellulase (S_CL) in non-rhizosphere was less than that in inter-rhizosphere (*p* < 0.05). Thus, there were differences in enzyme activities in soils from different habitats and also between inter- and non- rhizosphere in the same area.

### 3.5 Relationship between soil microbial communities and soil factors

**Figure 8.**
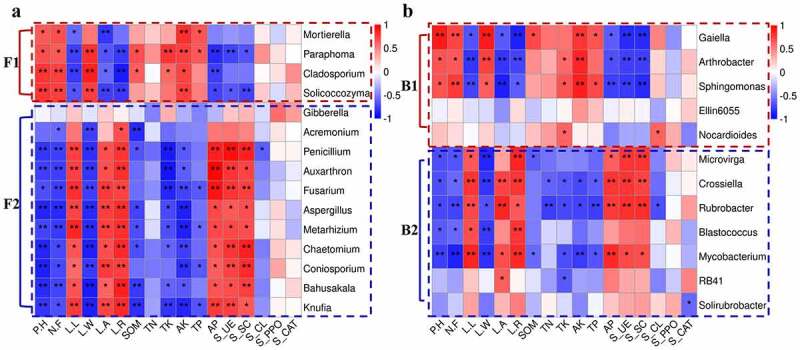
Spearman correlation heat map between soil microbial communities and environmental factors of *L.regale*. a: fungi; b: bacteria

The morphology of *L.regale* in different habitats varies significantly, and this variation is closely related to the characteristics of soil factors. Some studies have confirmed that soil physicochemical factors, soil enzyme activity and microbial community diversity and structure are closely related. Therefore, we selected common microorganisms (4 fungal genera and 5 bacterial genera) and differential microorganisms (11 fungal genera and 7 bacterial genera) at the genus level in both hillside and valley, and analyzed their spearman correlation with the phenotypic characteristics, soil physicochemical factors, and soil enzyme activity of *L.regale*, and then made a heat map using R (Figure 8). The results showed that the plant appearance morphology correlated significantly with most microorganisms. Among them, *Cladosporium* and *Solicoccozyma* in fungi and *Gaiella* in bacteria showed highly significant positive correlation with plant height and flower number (*p* < 0.01). At the same time, *Penicillium, Auxarthron, Aspergillus, Metarhizium, Bahusakala* in fungi, and *Mycobacterium* among bacteria showed highly significant negative correlations (*p* < 0.01) with Lily plant height and flower number, indicating that these microorganisms can affect Lily plant height and flower number.

The X and Y axes are environmental factors and abundant fungal and bacterial genera, respectively. The correlation coefficient (R) appears in different colors. The right side of the legend is the color range of different R values. The red color represents positive correlation and the blue color represents negative correlation. Significant values are shown as: * 0.01 < P ≤ 0.05; ** 0.001 < P ≤ 0.01. P.H: plant height; N.F: numbers of flowers; L.L: leaf length; L.W: leaf width; L.A: leaf aera; L.R: leaf aspect ratio. SOM: soil organic matter; TN: total nitrogen; TK: total potassium; AK: available potassium; TP: Total phosphorus; AP: available phosphorous; S_UE: soil urease; S_SC: soil sucrase; S_CL: soil cellulase; S_PPO: Soil polyphenol oxidase; S_CAT: soil catalase.

Among the soil physicochemical factors, available potassium (AK) and available phosphorous (AP) were more correlated with microorganisms, and total phosphorus (TP) and total nitrogen (TN) were less correlated. Among the fungi, *Paraphoma, Mortierella, Solicoccozyma, Knufia, Fusarium, Aspergillus, Metarhizium, Chaetomium* and *Coniosporium* were highly significantly correlated with soil AK (*p* < 0.01), while *Paraphoma, Cladosporium, Penicillium, Auxarthron, Knufia* and *Fusarium* were highly significantly correlated with AP (*p*< 0.01) (Figure 8(a)). Among the bacteria, *Gaiella, Arthrobacter, Sphingomonas* and *Mycobacterium* were highly significantly correlated with soil AK (*p* < 0.01), while *Sphingomonas, Crossiella, Rubrobacter* and *Mycobacterium* were highly significantly correlated with AP (*p* < 0.01). Among the soil enzymes, those with greater correlation with microorganisms were S_UE and S_SC, while the other enzymes did not correlate well with microorganisms. Among the fungi, *Penicillium, Fusarium, Chaetomium* and *Conisporium* showed highly significant positive correlations with soil S_UE and S_SC (*p* < 0.01). Among the bacteria, *Arthrobacter, Gaiella* and *Sphingomonas* showed highly significant negative correlations with soil S_UE and S_SC (*p* < 0.01), while *Crossiella, Microvirga* and *Rubrobacter* showed highly significant positive correlations with soil S_UE and S_SC (*p* < 0.01).

From Figure 8, it showed that both fungal and bacterial communities can be clustered into two groups. We named F1 and F2 for the fungal group, and B1 and B2 for the bacterial group. F1 and B1 were positively correlated with plant height, flower number, leaf width, and AK, and negatively correlated with leaf length, leaf area, leaf aspect ratio, AP, S_UE, and S_SC of *L.regale*, while F2 and B2 showed diametrically opposite correlation trends with these factors. Thus, representative microorganisms in the soils of *L.regale* from different habitats formed two types of groups, and their correlation characteristics with plant phenotypic characteristics and soil physicochemical factors and soil enzymes were diametrically opposed, suggesting that there may be some structural characteristics.

## 4. Disscusion

Lily is a famous horticultural plant with great ornamental value and it is one of the top five cut flower species in the world [25], enjoying immense popularity in the international flower market. At present, the various cultivars of Lily and their original relatives or hybrids can be divided into nine categories, such as Asiatic Lily hybrids, European Lily, trumpet hybrids and Orelian hybrids, among which *L.regale* is important genetic breeding parental resources of trumpet Lily, with horticultural characteristics of large and numerous flowers and tall plants [26]. There are considerable differences in the morphological of *L.regale* in different habitats in the Wenchuan area. The diversity and complexity of horticultural characters are closely related to plant genetic characteristics, soil factors and microbes. The interactions between plant and soil microbiome regarded as the second genome of plants, are extremely complex [27–29]. Therefore, studying on the relationship between soil microbes of *L.regale* and this horticultural phenotypic trait can identify related microorganisms.

Currently, many studies on soil microbiology focused on the diversity and abundance of soil microorganisms, the search for dominant or harmful microorganisms, their correlations with soil physicochemical factors, and the effects on plants [30–33]. For example, Sousa’s study [34] showed that TN and K are soil physicochemical factors, having high correlations with microorganisms in soil. The *Chloroflexi* significantly positively correlated with TN, and *Actinobacteria, Bacteroidetes, Chloroflexi, Patescibacteria*, and *Planctomycetes* significantly positively correlated with K. A study by Shen et al. also showed that TN were significantly correlated with some bacterial phyla (e.g. *Acidobacteria*, Chloroflexi). *Acidobacteria* was positively correlated with TN, and *Chloroflexi* was negatively correlated with TN [35]. Balbontin et al found that *Salmonella typhimurium* and *Aspergillus niger* had an effect on the height of maize plants, while co-inoculation of maize plants with *Salmonella typhimurium* and *Aspergillus niger* resulted in a significant reduction in plant height compared to their inoculation with *Salmonella typhimurium* or *Aspergillus niger* alone [36]. In this study, we selected *L.regale* located in the arid valley of the upper Minjiang River, a conservative habitat, where there is a lack of anthropogenic and other disturbing factors and thus this habitat is conducive to the identification of microorganisms associated with growth and its phenotype. We found that microorganisms correlated with the number of flowers and the high performance of the plant, such as *Cladosporium, Solicoccozyma, Gaiella*. Verbon et al. found that *Pseudomonas simiae* induced iron uptake, stimulated plant growth and increased plant height and fresh weight of above-ground parts of *Arabidopsis thaliana* when iron was sufficient in the soil [37]. However, most of these studies concentrated on just one or a few microorganisms with high correlation. Plant height and flower number are important selection targets for new Lily varieties, so we can elaborate various kinds of EM (effective microorganisms) such as flower-rich EM, plant-high EM, etc., to satisfy different needs according to the microorganisms associated with them in subsequent studies.

We further analyzed the whole correlated microorganisms and found an interesting phenomenon that the common and differential microbial communities in soils of *L.regale* clustered into two distinct groups based on positive and negative correlations. One group was positively correlated with the number of flowers, plant height, and leaf width of *L.regale*, and the other group was positively correlated with the leaf length, leaf area, and leaf aspect ratio of *L.regale*. The finding suggested that there may be structural characteristics of representative microorganisms in wild habitat soils, and the correlation between these two groups with soil physicochemical properties and enzyme activities showed a similar picture. Lily is a bulbous perennial herbaceous plant with relatively stable rhizosphere soil microorganisms and structure, suggesting that the structural characteristics of this dominant community may influence the phenotype of Lily. Thus, the structure and diversity of the rhizosphere microbial community may drive different plant phenotypes. The study of the original species of horticultural plants using this method is a scientific guide for the domestication and breeding of horticultural plants.

## 5. Conclusion

Phenotypic plasticity of plants refers to different phenotypic characteristics by plants caused by variable environments. It is the adaptation and expression of plants to their environment. Currently, it has grown up to be an important research field in ecological and horticulture. In this study, the soil microbiome and environmental factors of *L.regale* in different habitats were investigated, and the results showed that the structure and diversity of rhizosphere microbiome may drive different plant phenotypes. The application of this method to study the original species of horticultural plants is a systematic guide to the domestication and breeding of horticultural plants. The highly variable topography of the peripheral Sichuan Basin has produced a rich diversity of flowering plant resources. However, horticulture is diverse and complex not only in terms of plants, but also in terms of soils and microorganisms. Further study of the relationship between Microecology and Epigenetics will provide original plant species for horticulture, and improve the utilization of microbial biofertilizers for horticulture.
